# Metabolic engineering of riboflavin production in *Ashbya gossypii* through pathway optimization

**DOI:** 10.1186/s12934-015-0354-x

**Published:** 2015-10-14

**Authors:** Rodrigo Ledesma-Amaro, Cristina Serrano-Amatriain, Alberto Jiménez, José Luis Revuelta

**Affiliations:** Departamento de Microbiología y Genética, Universidad de Salamanca, Campus Miguel de Unamuno, 37007 Salamanca, Spain

**Keywords:** Riboflavin, *Ashbya gossypii*, *RIB*, *ADE12*, Metabolic engineering

## Abstract

**Background:**

The industrial production of riboflavin mostly relies on the microbial fermentation of flavinogenic microorganisms and *Ashbya gossypii* is the main industrial producer of the vitamin. Accordingly, bioengineering strategies aimed at increasing riboflavin production in *A. gossypii* are highly valuable for industry.

**Results:**

We analyze the contribution of all the *RIB* genes to the production of riboflavin in *A. gossypii*. Two important metabolic rate-limiting steps that limit the overproduction of riboflavin have been found: first, low mRNA levels of the *RIB* genes hindered the overproduction of riboflavin; second, the competition of the AMP branch for purinogenic precursors also represents a limitation for riboflavin overproduction. Thus, overexpression of the *RIB* genes resulted in a significant increase in riboflavin yield. Moreover, both the inactivation and the underexpression of the *ADE12* gene, which controls the first step of the AMP branch, also proved to have a positive effect on riboflavin production. Accordingly, a strain that combines both the overexpression of the *RIB* genes and the underexpression of the *ADE12* gene was engineered. This strain produced 523 mg/L of riboflavin (5.4-fold higher than the wild-type), which is the highest titer of riboflavin obtained by metabolic engineering in *A. gossypii* so far.

**Conclusions:**

Riboflavin production in *A. gossypii* is limited by a low transcription activity of the *RIB* genes. Flux limitation towards AMP provides committed substrate GTP for riboflavin overproduction without detrimental effects on biomass formation. A multiple-engineered *Ashbya* strain that produces up to 523 mg/L of riboflavin was generated.

**Electronic supplementary material:**

The online version of this article (doi:10.1186/s12934-015-0354-x) contains supplementary material, which is available to authorized users.

## Background

Riboflavin (vitamin B2) is an essential nutrient for humans and animals that must be obtained from the diet. Accordingly, it is usually included in fortified foods and multivitamin supplements [1]. The production of riboflavin by microbial fermentation is a paradigmatic example of white biotechnology using different cell factories such as *Bacillus subtilis*, *Candida flareri* and, especially, *Ashbya gossypii* [2]. Indeed, nearly half the world production of riboflavin (8000 tons per year) is obtained through the fermentation of *A. gossypii* strains [3].

*Ashbya gossypii* is a filamentous hemiascomycete that overproduces riboflavin naturally. Thus, industrial strains of *A. gossypii* for riboflavin production have been obtained by a combination of random chemical mutagenesis with metabolic engineering of the purine pathway [1]. The development of rational approaches for the manipulation of *A. gossypii* is now supported with novel genomic tools, including a genome-scale metabolic model of *A. gossypii* (iRL766) [4]. In addition, new strains of *A. gossypii* for the production of folic acid and single-cell oil have been described recently [5, 6].

**Fig. 1:**
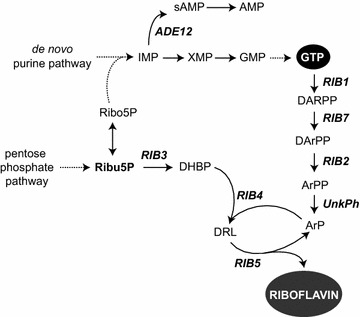
Diagram of the riboflavin biosynthetic pathway. The genes manipulated in this work are indicated in *bold*. *Dashed lines* indicate multistep reactions. *Ribu5P* ribulose-5-phosphate; *ribo5P* ribose-5-phosphate; *DARPP* 2,5-diamino-6-ribosylamino-4(3H)-pyrimidinedione phosphate; *DArPP* 2,5-diamino-6-ribitylamino-4-(3*H*)-pyrimidinedione-5′-phosphate; *ArPP* 5-amino-6-ribitylamino-2,4-(1*H*,3*H*)-pyrimidinedione-5′-phosphate; *ArP* 5-amino-6-ribitylamino-2,4-(1*H*,3*H*)-pyrimidinedione; *DHBP* 3,4-dihydroxy-2-butanone-4-phosphate; *DRL* 6,7-dimethyl-8-ribityl-lumazine

The biosynthesis of riboflavin is initiated from two main precursors: GTP and ribulose-5-phosphate (Ribu5P) (Fig. 1). GTP is synthesized from the purine pathway and its availability has been shown to be important for riboflavin production. In this regard, the increase in metabolic flux towards the synthesis of GTP results in the overproduction of riboflavin [7–9]. GTP and Ribu5P are transformed into riboflavin via a 7-reaction pathway that is controlled by the *RIB* genes [10] (Fig. 1). There are two branches that converge to form 6,7-Dimethyl-8-ribityl-lumazine (DRL). One branch synthesizes 5-Amino-6-ribitylamino-2,4-(1*H*,3*H*)-pyrimidinedione (ArP) from GTP through four consecutive reactions catalyzed by the enzymes RIB1, RIB7, RIB2 and an unknown phosphatase (Fig. 1). On the other branch, RIB3 transforms Ribu5P into 3,4-Dihydroxy-2-butanone-4-phosphate (DHBP). Then, both ArP and DHBP are conjugated by RIB4 to produce DRL, which is subsequently converted into riboflavin by the product of the *RIB5* gene (Fig. 1). All the *RIB* genes have been identified in *A. gossypii* [11] but so far their contribution to riboflavin overproduction has not been fully characterized.

In *A. gossypii* riboflavin is mainly produced during the production phase, when the growth rate decreases. In contrast, exponential growth occurs during the trophic phase, when riboflavin yield is minimal [10]. Accordingly, riboflavin biosynthesis is tightly regulated at both the enzymatic and transcriptional levels as previously described both for the purine and RIB pathways in *A. gossypii* [7, 9, 11]. In this regard, transcriptional regulation occurred during the riboflavin production phase has been predicted by a genome-scale metabolic reconstruction, thus revealing a number of target genes that can be considered as good candidates for metabolic engineering approaches to increase the production of riboflavin. Interestingly, the *RIB2*, *RIB3*, *RIB4* and *RIB5* genes were predicted to be up-regulated genes during riboflavin overproduction in *A. gossypii* [4]. In good agreement, several reports have also shown a relationship between the transcriptional regulation of *RIB* genes and an increase of the riboflavin biosynthesis in several organisms [12–14].

As mentioned above, riboflavin overproduction has been achieved through the deregulation of the purine pathway at different levels to increase the pool of GTP [7–9]. It is noteworthy that both the GMP and AMP branches of the purine pathway compete for the common precursor IMP. Thus, in *C. famata* (*C. flareri*), the heterologous expression of both the transcription factor *SEF1* and the purinogenic enzyme IMP dehydrogenase from *Debaryomyces hansenii*, together with the overexpression of the *RIB1* and *RIB7* genes, resulted in a 4.1-fold increase in riboflavin production [15]. Hence, a limiting riboflavin biosynthetic pathway may mask the identification of new mutations improving the production of riboflavin.

The objective of this work was to study the relative contribution of each *RIB* gene to the riboflavin production process in *A. gossypii*. Accordingly, the transcriptional pattern of the *RIB* genes was analyzed and the potential impact of each *RIB* gene on the metabolic control of riboflavin production was determined by overexpression of single *RIB* genes as well as by combining the overexpression of up to five *RIB* genes to deregulate the entire pathway. In addition, the performance of a deregulated strain overexpressing the full set of *RIB* genes was challenged to support higher riboflavin productivity in response to a major availability of GTP substrate. This was achieved by metabolic flux re-direction towards GMP by reducing the synthesis of AMP from IMP. In summary, we found that both the availability of GTP substrate from the purine pathway and the transcriptional activity of the *RIB* genes are rate-limiting steps for riboflavin overproduction.

## Results

### Transcriptional analysis of the *RIB* genes in *A. gossypii*

As previously described, several reports have established a relationship between the transcriptional regulation of some of the *RIB* genes and the increase of riboflavin production [11–14]. In addition, the analysis of metabolic flux changes related to the production of riboflavin using the iRL766 model predicted the up-regulation of most of the *RIB* genes during the production phase in *A. gossypii* [4]. Accordingly, we were prompted to determine the RNA levels of all the six *RIB* genes encoded in the genome of *A. gossypii*.

**Fig. 2:**
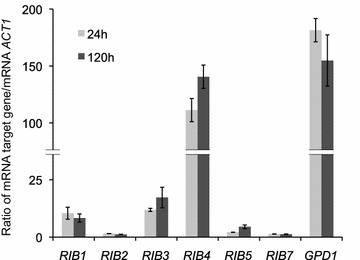
Quantitative-real time PCR of the *RIB* genes in *A. gossypii*. Relative transcription levels of the *RIB* genes in our wild-type strain of *A. gossypii* during both the trophic phase (24 h, exponential growth) and the production phase (120 h, stationary growth). The *GPD1* gene was included as a control. Transcription levels were normalized using the *A. gossypii ACT1* gene as a reference. The results are the means of two independent experiments performed in duplicate and are expressed as a ratio of the cDNA abundances of the target genes with respect to the *ACT1* mRNA levels

The transcriptional analysis of the *RIB* genes was carried out by qRT-PCR. Total RNA from *A. gossypii* MA2 cultures in both the trophic phase (24 h of culture) and the production phase (120 h of culture) were obtained. Quantitative analysis of the *RIB* genes in cDNA samples was carried out using the housekeeping gene *ACT1* as a reference [6]. With the exception of *RIB4*, the level of RNA for all the *RIB* genes was very low in both the trophic and production phases; ranging from 1–5  (for *RIB2*, *RIB5* and *RIB7*) to 10–15 % (for *RIB1* and *RIB*3) of the transcript level of the moderately transcribed gene *ACT1* (Fig. 2). *RIB4* showed a high transcriptional level, similar to that of the *GPD* gene (Fig. 2), whose promoter is widely used for gene overexpression in *A. gossypii* [9]. In contrast to the prediction of the iRL766 model, the transcription rate of the *RIB* genes showed no or only minor increments during the production phase (Fig. 2).

### Single gene overexpression of the *RIB* genes in *A. gossypii*

The results reported above revealed that the transcription of most of the *RIB* genes remains low throughout the trophic and production phases of *A. gossypii* culture. Accordingly we wished to determine the effect of the overexpression of each *RIB* gene on riboflavin production in *A. gossypii*. The *RIB4* was excluded from the overexpressions experiments, since this gene already showed a high transcriptional level in the wild-type.

**Fig. 3:**
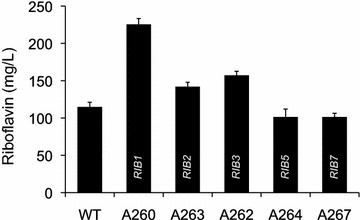
Riboflavin production after the overexpression of *RIB* genes in *A. gossypii*. Quatification of riboflavin production in the *A. gossypii* strains that overexpress one of the *RIB* genes. The data are the means of three independent experiments performed in duplicate

Gene overexpressions were carried out using an integrative cassette harboring a geneticin-resistance selection marker and the strong promoter *P*_*AgGPD*_. The cassette was flanked by recombinogenic sequences for each *RIB* gene and was integrated by homologous recombination to replace the native promoter region of each gene. Overexpression of the *RIB* genes was verified by qRT-PCR analysis (Additional file 1: Fig. S1a) and the riboflavin production of the mutant strains was determined. As shown in the Fig. 3, the overexpression of *RIB1*, *RIB2* or *RIB3* triggered an increase in riboflavin production. The overexpression of either *RIB1* or *RIB3*, which code for the first enzymes of each branch in the riboflavin pathway, induced the highest increases in riboflavin production. In contrast, the overexpression of either *RIB5* or *RIB7* did not cause any significant change in the production of riboflavin (Fig. 3).

### Global overexpression of the *RIB* genes in *A. gossypii*

**Fig. 4:**
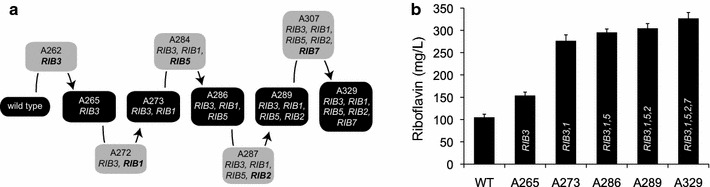
Overexpression of the *RIB* genes in a single strain of *A. gossypii*.** a** Pipeline of the construction of the A329 strain and** b** quatification of riboflavin production in the *A. gossypii* strains. The data are the means of three independent experiments performed in duplicate

We observed that overexpression of some of the *RIB* genes induced an increase in riboflavin production in *A. gossypii*. Thus, the low transcription rate of the *RIB* genes may be a limiting-rate step for riboflavin overproduction and, consequently, a maximal yield of riboflavin might be obtained with the overexpression of all the *RIB* genes. Accordingly, it was decided to overexpress all the *RIB* genes to quantify the riboflavin production in a quintuple overexpressing strain. A *loxP*-*KanMX*-*loxP* selection marker was used for gene overexpression. The *loxP* repeated inverted sequences enabled the selection marker to be eliminated, and subsequently reused, by expressing a Cre recombinase after each round of transformation, as described elsewhere [6]. The gene overexpression pipeline is depicted in Fig. 4a. The A329 strain, which overexpresses the *RIB1*, *RIB2*, *RIB3*, *RIB5* and *RIB7* genes, was obtained after 10 transformations either to integrate the *P*_*AgGPD*_ into the target loci or to remove the *KanMX* selection marker (Fig. 4a). The overexpression of the *RIB* genes in the new mutant strains was verified by qRT-PCR (Additional file 1: Fig. S1b).

Riboflavin production was measured in the quintuple overexpressing strain and also in all the intermediate strains (Fig. 4b). As shown above, the overexpression of *RIB3* alone triggered a remarkable increase in riboflavin production (46 % more riboflavin than the WT strain). In addition, the combination of the overexpression of both *RIB1* and *RIB3* (strain A273) increased the riboflavin yield by more than 2.5-fold in comparison with the WT. Moreover, the overexpression of *RIB5*, *RIB2* and *RIB7* caused an additional increase in riboflavin production with respect to the A273 strain. Overall, the A329 strain, which simultaneously overexpressed the *RIB1*, *RIB2*, *RIB3*, *RIB5* and *RIB7* genes, showed the highest level of riboflavin production (326.6 mg/L), representing a 3.1-fold increase over the WT production (Fig. 4b).

### Flux balance analysis of the purine pathway for riboflavin production

In the absence of enzymatic regulation constrains, the strain containing the overexpressed, deregulated riboflavin biosynthetic pathway should not be limited in the riboflavin pathway itself, but rather it should be able to respond to increases in the pool of the GTP substrate by efficiently increasing the rate of riboflavin product formation. To assess this hypothesis we decided to increase the GTP supply available for riboflavin synthesis and determine the effect on riboflavin production in both the WT and RIB-overexpressing (A329) strains.

The purine pathway provides GTP, which is one of the main precursors of riboflavin. Since our previous results demonstrated that the deregulation of the purine pathway induces a significant increase of riboflavin overproduction in *A. gossypii* [7–9], an increase in the intracellular pool of GTP could be correlated with a higher riboflavin yield in *A. gossypii*. IMP is the central metabolite of the purine pathway, which can subsequently be converted either to AMP or GMP via a two-branch pathway (Fig. 1). Thus, IMP metabolic flux towards GMP/GTP production compete with the AMP branch, which consists of two enzymatic reactions controlled by adenylosuccinate synthase (*ADE12*) and adenylosuccinate lyase (*ADE13*). Indeed, flux balance analysis of riboflavin production using the *A. gossypii* iRL766 model predicted that the AMP branch of the purine pathway is down-regulated during the production phase, while the GMP branch, leading to GTP and thereafter riboflavin, is not transcriptionally regulated. These results suggest that a control over the IMP hub affects riboflavin production.

Accordingly, we used the *A. gossypii* iRL766 model to simulate a reduction in metabolic flux towards the AMP branch by decreasing the activity of the Ade12 enzyme (Fig. 1). The in silico simulation comprised 316 metabolic reactions, including enzymatic cofactors that could affect metabolic flux exchange in both the purine and riboflavin pathways. The output of the model predicted a linear increase in riboflavin production that was directly correlated with the reduction in Ade12 activity (Additional file 2: Fig. S2). Thus, in order to increase the supply of GTP available for riboflavin synthesis in *A. gossypii*, the *ADE12* gene can be considered a solid candidate for metabolic engineering through deletion. In addition, engineering the IMP metabolic hub can serve to analyze the performance of a deregulated, overexpressed riboflavin pathway in response to a greater availability of guanine precursors.

### Metabolic engineering of the *IMP* hub gene in *A. gossypii*

To analyze the effect of the *ADE12* gene-deletion on riboflavin production in *A. gossypii* a gene knockout strain was constructed using a *loxP*-*KanMX*-*loxP* selection marker flanked by *ADE12*-flanking recombinogenic sequences, as described above (see “Methods” section). As expected, the *ade12∆* strain was viable but showed adenine auxotrophy. Analysis of riboflavin production revealed that the *ade12∆* strain was able to produce up to 246 mg/L of riboflavin, which represents a 2.5-fold increase in riboflavin production compared to the wild-type strain. In this sense, our results confirm the idea of GTP availability as a limiting step for riboflavin overproduction.

**Fig. 5:**
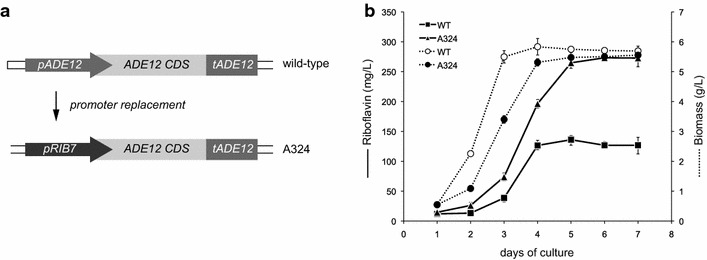
Underexpression of the *ADE12* gene in *A. gossypii*. **a** Diagram of the promoter replacement of the *ADE12* gene and **b** biomass and riboflavin production analyses after the underexpression of the *ADE12* gene in *A. gossypii*. The data are the means of three independent experiments performed in duplicate

Although the deletion of *ADE12* triggers the production of riboflavin, the presence of auxotrophic mutations in the strains is highly undesirable for large-scale industrial fermentations. Therefore, we decided to analyze the effect of *ADE12* gene underexpression on riboflavin production. As shown above, the *RIB7* gene has very low levels of mRNA in the WT strain. Indeed, a qRT-PCR analysis revealed that the transcription rate of *RIB7* was approximately 62-fold lower than that of the *ADE12* gene. Accordingly, the promoter sequence of the *RIB7* gene *(P*_*RIB7*_) was used for *ADE12* gene underexpression. Thus, the *ADE12* native promoter was replaced by *P*_*RIB7*_ using a *loxP*-*KanMX*-*loxP* selection marker, which was subsequently eliminated as described above (Fig. 5a). The promoter replacement was confirmed both by DNA sequencing of the genomic amplicon (data not shown) and by qRT-PCR analysis. As expected, the mRNA levels of *ADE12* were extremely reduced in the *P*_*RIB7*_-*ADE12* strain (approximately 70-fold lower than those in the wild-type strain). However, this transcription level was sufficient to sustain the growth of the mutant in MA2 media without adenine supplementation. Moreover, the underexpression of *ADE12* did not significantly affect biomass formation and riboflavin production was similar to that of the *ade12∆* strain (Fig. 5b).

### Engineering the metabolism of purines and overexpression of the *RIB* genes trigger riboflavin overproduction

As shown above, the supply of guanine precursors and the transcription of the *RIB* genes largely determine the riboflavin yield in *A. gossypii*. Accordingly, in order to analyze the a riboflavin overproducer strain, we decided to combine both the overexpression of the *RIB* genes and the underexpression of the *ADE12* gene.

**Fig. 6:**
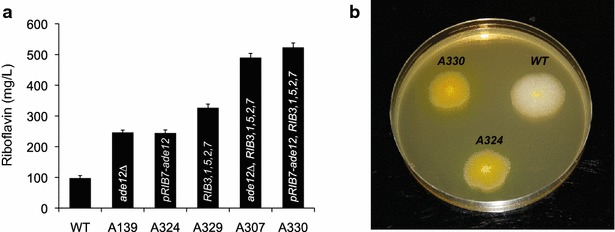
Riboflavin overproduction in the engineered *A. gossypii*. **a** quatification of riboflavin production in *A. gossypii* strains. The data are the means of three independent experiments performed in duplicate. **b** solid MA2 plates of the strains A330, A324 and wild-type of *A. gossypii*. The strain A330 that was modified both for the underexpression of the *ADE12* gene and for the overexpression of five *RIB* genes afforded the highest riboflavin yield (in *yellow*)

The quintuple *RIB*-engineered strain A329, which overexpresses the *RIB1*, *RIB2*, *RIB3*, *RIB5* and *RIB7* genes, was transformed with either the *ade12Δ* deletion cassette or the *P*_*RIB7*_ cassette to replace the *ADE12* native promoter. The genomic integrations of each module were confirmed both by analytical PCR and qRT-PCR to test *ADE12* mRNA (data not shown). Other parameters, such as the growth rate, biomass production and sporulation ability, were analyzed but no significant differences were found between these new strains and the wild-type strain (data not shown). Finally, a marked increase in riboflavin production was found when these two approaches were conjugated in a single strain (Fig. 6). A total yield of 523 mg/L of riboflavin was obtained when the overexpression of *RIB* genes and the underexpression of *ADE12* were combined. This riboflavin level in our mutant strain represents an increase of 5.4-fold with respect to production by the wild-type strain (Fig. 6).

## Discussion

Riboflavin production by microbial fermentation is a paradigm of a biotechnological process that has replaced chemical synthesis in the industry. Although different microorganisms are used for the microbial production of riboflavin, *A. gossypii* has proved to be one of the most effective producers for industry [2, 16]. Accordingly, the metabolic engineering of *A. gossypii* for riboflavin overproduction represents an important challenge for research in biotechnology.

The transcription of the *RIB* genes in *A. gossypii* has been analyzed during both the trophic and production phases. We found that the *RIB4* gene was highly transcribed, mainly during the production (stationary) phase, which might indicate the presence of an inducible promoter within the *RIB4* sequence. In fact, according to previously published transcriptomic data, *RIB4* is the 28th most abundant RNA during the stationary phase in *A. gossypii* [17]. Therefore, the *RIB4* promoter could be an interesting genetic tool for the overexpression of target proteins during the stationary phase in *A. gossypii*. In contrast to *RIB4*, the *RIB2* and *RIB7* genes were very poorly transcribed during *A. gossypii* growth and production phases. Indeed, the substitution of the *ADE12* native promoter by the *RIB7* promoter was sufficient to minimize the activity of *ADE12* and the metabolic flux towards AMP synthesis. This represents proof of concept for the use of the *RIB7* promoter for gene underexpression in *A. gossypii*. This strategy may be very useful as a new molecular tool both to prevent undesired auxotrophies in industrial strains and also to study the effect of the downregulation of essential genes.

We found that overexpression of *RIB1* and *RIB3* (either single or combined overexpression) triggered a high increase in riboflavin yield, which indicates that the first steps of each branch of the riboflavin biosynthetic pathway (governed by the *RIB1* and *RIB3* genes) are important control points for riboflavin production. Nevertheless, the quintuple overexpressing strain rendered the highest level of riboflavin production, affording 326.6 mg/L. All together, these results suggest that all the *RIB* genes are coordinately expressed so that the control of riboflavin production in *A. gossypii* is more or less evenly distributed over all the genes and enzymes of the RIB pathway and lacks strong controlling individual steps.

It has been previously shown that increasing the pool of metabolic precursors from the purine pathway also induces a significant overproduction of riboflavin in *A. gossypii* [7–9, 18]. In this regard, an in silico analysis of the biosynthesis of riboflavin using the genome-scale metabolic model iRL766 [4] predicted an increase in riboflavin production by reducing the competing branch leading to AMP synthesis. Accordingly, the deletion or underexpression of *ADE12* caused an enhancement of riboflavin accumulation, thus supporting the idea that the availability of the immediate precursor of riboflavin biosynthesis is another metabolic restriction for riboflavin overproduction.

The overexpression of the *RIB* genes ensures that the cell is devoid of bottlenecks in the riboflavin biosynthetic pathway. This provides a cell model system to identify new mutations or target genes improving riboflavin production that otherwise would be masked by a limiting riboflavin biosynthetic pathway. The overexpression of the riboflavin pathway is also a strategy of great value for further improvement of current industrial strains because it is highly unlikely that those strains may have accumulated all the necessary mutations required for overexpression of all six *RIB* genes. In this regard, we found that the underexpression of the *ADE12* gene has an additive effect over the overexpression of five *RIB* genes, providing the highest yield of riboflavin production obtained by metabolic engineering in *A. gossypii* so far. According to our results, metabolic engineering of the IMP hub and improvement of the riboflavin biosynthetic pathway may circumvent two important restraints limiting production of riboflavin in *A. gossypii*. We believe that iterative metabolic engineering in combination with fluxomics could help drive forward both our understanding of riboflavin metabolism in *A. gossypii* and the generation of new strains with a higher riboflavin production capacity.

## Conclusions

We used metabolic engineering to combine the overexpression of all the *RIB* genes with the enhancement of the GMP purine branch to generate a strain (A330) that produces 5.4-fold more riboflavin than the wild-type strain (523 mg/L). We foresee that this strategy will provide a scalable and controllable way to increase the industrial production of this economically important vitamin.

## Methods

*Ashbya gossypii strains, media, and growth conditions.* The *A. gossypii* ATCC 10895 strain was used and considered a wild-type strain. Other *A. gossypii* strains used in the study are listed in the Additional file 4: Table S2. *A. gossypii* was cultured at 28 °C using either MA2 rich medium or synthetic minimal medium lacking adenine (SC-ade) [9]. *A. gossypii* transformation, its sporulation conditions and spores isolation were as previously described [9, 19].

*Quantitative Real*-*Time PCR.* Quantitative real-time PCR (qRT-PCR) was performed with a LightCycler 480 real-time PCR instrument (Roche), using SYBR green I master mix (Roche) and following the manufacturer’s instructions. Total RNA samples were obtained as described previously [7] and cDNA samples were prepared using the Transcriptor First Strand cDNA Synthesis Kit (Roche). Primer sequences are indicated in the Additional file 4: Table S2. All real-time PCR reactions were performed in duplicate and in at least two independent experiments. Quantitative analyses were carried out using the LightCycler 480 software. Quantitative values were obtained as the threshold PCR cycle number (*Ct*) when the increase in the fluorescent signal of the PCR product showed exponential amplification. The target gene mRNA level was normalized to that of Ag*ACT1* in the same sample. The relative transcription level of the target gene was calculated using the 2^−∆∆*Ct*^ method [20].

*Gene overexpression and gene deletion.* For gene overexpression_*AgGPD*_, the promoter sequence of the *AgGPD* gene was integrated upstream of the ATG initiator codon of each gene. An overexpression cassette comprising the *AgGPD* promoter (*P*) and the *loxP*-*KanMX*-*loxP* selectable marker, conferring resistance to geneticin (G418), was PCR-amplified using specific primers for each gene (Additional file 4: Table S2). The overexpression modules were used to transform spores of *A. gossypii* and positive clones were selected in G418-containing medium. Homokaryon clones were obtained by sporulation of the primary transformants. The correct genomic integration of each overexpression cassette was confirmed by analytical PCR followed by DNA sequencing. Gene overexpression was checked by qRT-PCR analysis. For the deletion of *AgADE12*, a gene replacement cassette was constructed for the *ADE12* gene by PCR amplification of the *loxP*-*KanMX*-*loxP* marker (see primer sequences in the Additional file 4: Table S2). The replacement cassette was used to transform spores of *A. gossypii.* Primary transformants were selected in G418-containing medium and homokaryon clones were isolated by sporulation of the primary transformants. The homologous integration of the replacement cassette was confirmed by analytical PCR followed by DNA sequencing.

*Determination of riboflavin production.* Riboflavin was measured using a spectrophotometric method. Briefly, a volume of 1 mL of the culture was harvested and mixed with 1 mL of 0.1 N HCl. The suspension was heated at 100 °C for 30 min. Then, samples were cooled down and mycelia were broken using glass beads. The liquid phase containing both extracellular and intracellular riboflavin was recovered and used for riboflavin determination. A calibration curve was performed using pure riboflavin (Sigma-Aldrich), which was processed in an identical way to the samples. Riboflavin concentration was determined by reading the absorbance of each sample at 450 nm. Measurements were performed using a Varioskan microtiter plate reader (Thermo Scientific).

*In silico flux balance analysis*. Flux balance analysis calculations were performed using MATLAB (Mathworks, Natick, MA) and the Raven toolbox [21]. The iRL766 genomic scale model for *A. gossypii* was used [4]. To calculate the maximum theoretical yields, riboflavin production reaction ‘RFLAVxtO’ was set as the objective function and the system of linear equations was solved. Biomass and glucose uptake were constrained to 0.09 h^−1^ and 1 mmol/gh, respectively, while NH3, sulfate, phosphate and adenine remained opened. The flux through ADE12 was fixed to 0.0117 mmol/gh (the maximum theoretical rate) and reduced manually to simulate a reduction in its metabolic flux. The model simulates adenine utilization from the salvage purine pathway, which allows normal growth even when the *de novo* pathway for adenine biosynthesis is limited. The *on line* tool Biomet toolbox 2.0 (http://biomet-toolbox.org/) was used to check the flux balance analysis.

## Additional files

10.1186/s12934-015-0354-x Quantitative-real time PCR of *RIB* genes in *A. gossypii* mutants. A, relative transcription levels of the *RIB* genes in the strains containing overexpression modules for each *RIB* gene compared with the *WT* strain. B, relative transcription levels of the *RIB* genes both in the *WT* and the *A329* strains. Transcription levels were normalized using the *A. gossypii* *ACT1* gene as a reference. The results are means of two independent experiments performed in duplicate and are expressed as a ratio of the cDNA abundances of the target genes with respect to the *ACT1* mRNA levels.
10.1186/s12934-015-0354-x Correlation between the ADE12-catalyzed flux and riboflavin production in *A. gossypii*. The *A. gossypii* iRL766 model was used to predict the effect of the ADE12 flux reduction over riboflavin biosynthesis. See “Methods” for details.
10.1186/s12934-015-0354-x *A. gossypii* strains used in this study.
10.1186/s12934-015-0354-x List of primers used in this study.
