# Supplementary material for: Metabolic engineering of riboflavin production in Ashbya gossypii through pathway optimization
Source: Microb Cell Fact. 2015 Oct 14;14:163. doi: 10.1186/s12934-015-0354-x (PMC4605130; doi:10.1186/s12934-015-0354-x)
Supplement: Supplementary file 1 — 10.1186/s12934-015-0354-x Quantitative-real time PCR of RIB genes in A. gossypii mutants. A, relative transcription levels of the RIB genes in the strains containing overexpression modules for each RIB gene compared with the WT strain. B, relative transcription levels of the RIB genes both in the WT and the A329 strains. Transcription levels were normalized using the A. gossypii ACT1 gene as a reference. The results are means of two independent experiments performed in duplicate and are expressed as a ratio of the cDNA abundances of the target genes with respect to the ACT1 mRNA levels. [file 12934_2015_354_MOESM1_ESM.pdf]

A

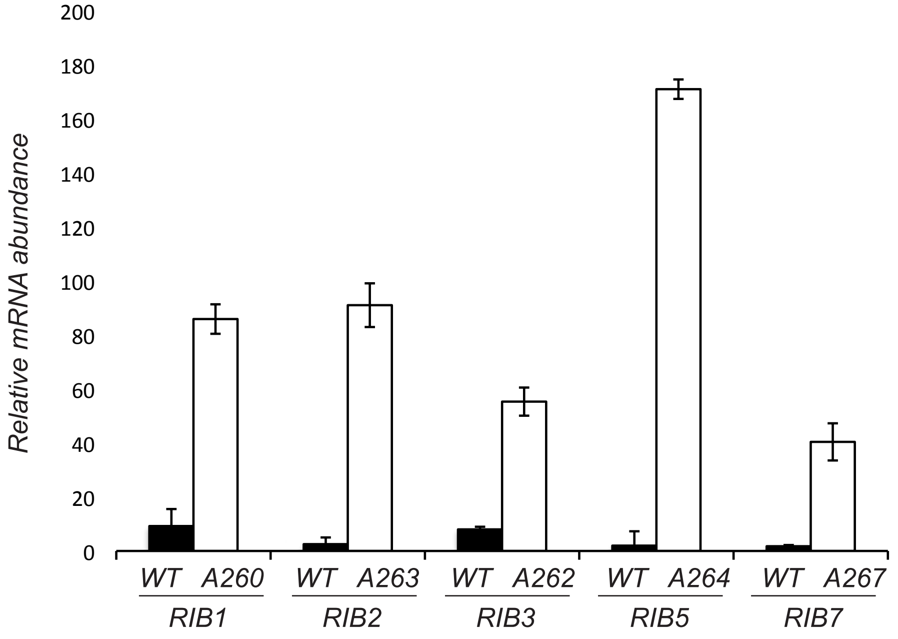

B

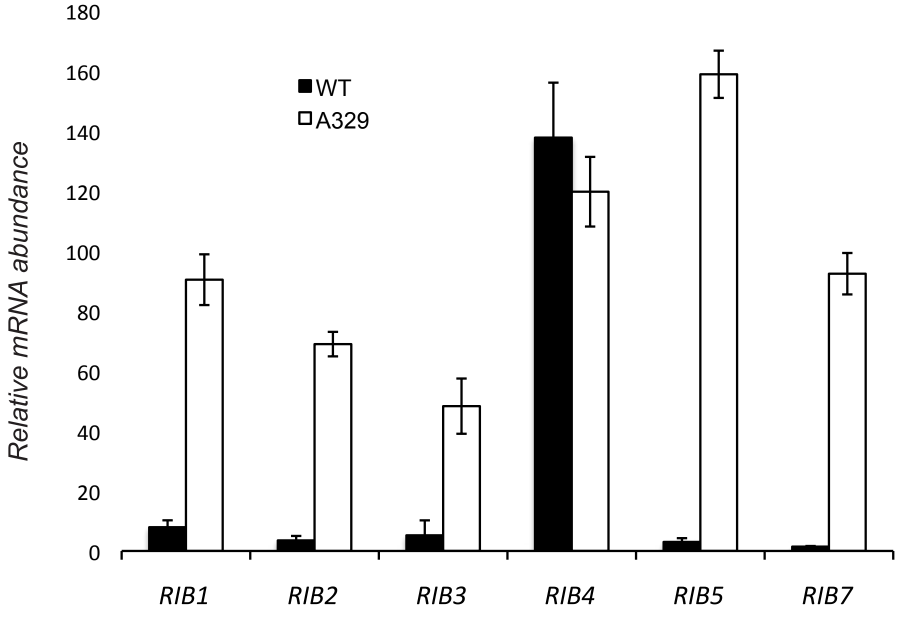

Additional File - Figure 1. Quantitative-real time PCR of *RIB* genes in *A. gossypii* mutants. A, relative transcription levels of the *RIB* genes in the strains containing overexpression modules for each *RIB* gene compared with the *WT* strain. B, relative transcription levels of the *RIB* genes both in the *WT* and the *A329* strains. Transcription levels were normalized using the *A. gossypii ACT1* gene as a reference. The results are means of two independent experiments performed in duplicate and are expressed as a ratio of the cDNA abundances of the target genes with respect to the *ACT1* mRNA levels.

Additional File - Figure 1
