# Supplementary material for: Metabolic engineering of riboflavin production in Ashbya gossypii through pathway optimization
Source: Microb Cell Fact. 2015 Oct 14;14:163. doi: 10.1186/s12934-015-0354-x (PMC4605130; doi:10.1186/s12934-015-0354-x)
Supplement: Supplementary file 2 — 10.1186/s12934-015-0354-x Correlation between the ADE12-catalyzed flux and riboflavin production in A. gossypii. The A. gossypii iRL766 model was used to predict the effect of the ADE12 flux reduction over riboflavin biosynthesis. See “Methods” for details. [file 12934_2015_354_MOESM2_ESM.pdf]

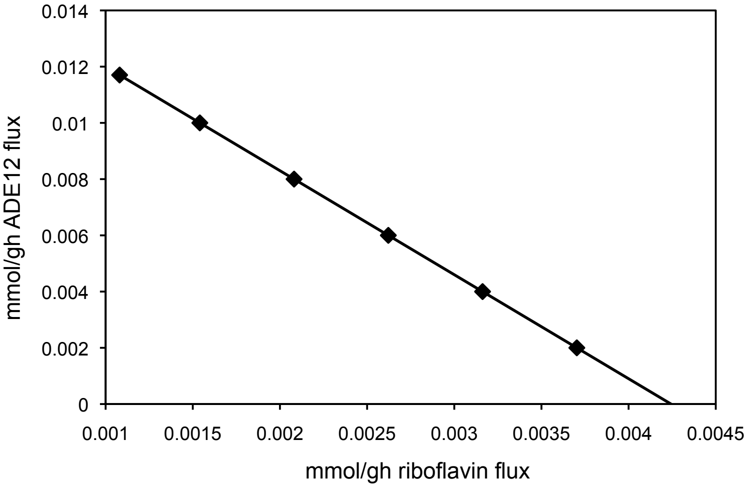

Additional File - Figure 2. Correlation between the ADE12-catalyzed flux and riboflavin production in *A. gossypii*. The *A. gossypii* iRL766 model was used to predict the effect of the ADE12 flux reduction over riboflavin biosynthesis. See Methods for details.

Additional File - Figure 2
