# Supplementary material for: Metabolic engineering of riboflavin production in Ashbya gossypii through pathway optimization
Source: Microb Cell Fact. 2015 Oct 14;14:163. doi: 10.1186/s12934-015-0354-x (PMC4605130; doi:10.1186/s12934-015-0354-x)
Supplement: Supplementary file 3 — 10.1186/s12934-015-0354-x A. gossypii strains used in this study. [file 12934_2015_354_MOESM3_ESM.docx]

| *Strain* | *Genotype* | *Phenotype* | *Source* |
| --- | --- | --- | --- |
| WT | wild type (ATCC 10895) | WT | our lab stock |
| *A139* | *ade12Δ* | ade^-^ | this work |
| *A260* | *loxP-KanMX4-loxP-P_GPD_-RIB1* | G418^R^ | “ |
| *A262* | *loxP-KanMX4-loxP-P_GPD_-RIB3* | G418^R^ | “ |
| *A263* | *loxP-KanMX4-loxP-P_GPD_-RIB2* | G418^R^ | “ |
| *A264* | *loxP-KanMX4-loxP-P_GPD_-RIB5* | G418^R^ | “ |
| *A265* | *P_GPD_-RIB3* | WT | “ |
| *A267* | *loxP-KanMX4-loxP-P_GPD_-RIB7* | G418^R^ | “ |
| *A272* | *P_GPD_-RIB3, loxP-KanMX4-loxP-P_GPD_-RIB1* | G418^R^ | “ |
| *A273* | *P_GPD_-RIB1, P_GPD_-RIB3* | WT | “ |
| *A284* | *P_GPD_-RIB1, P_GPD_-RIB3, loxP-KanMX4-loxP-P_GPD_-RIB5* | G418^R^ | “ |
| *A286* | *P_GPD_-RIB1, P_GPD_-RIB3, P_GPD_-RIB5* | WT | “ |
| *A287* | *P_GPD_-RIB1, P_GPD_-RIB3, P_GPD_-RIB5, loxP-KanMX4-loxP-P_GPD_-RIB2* | G418^R^ | “ |
| *A289* | *P_GPD_-RIB1, P_GPD_-RIB2, P_GPD_-RIB3, P_GPD_-RIB5* | WT | “ |
| *A307* | *P_GPD_-RIB1, P_GPD_-RIB2, P_GPD_-RIB3, P_GPD_-RIB5, loxP-KanMX4-loxP-P_GPD_-RIB7* | G418^R^ | “ |
| *A324* | *P_RIB7_-ADE12* | WT | “ |
| *A329* | *P_GPD_-RIB1, P_GPD_-RIB2, P_GPD_-RIB3, P_GPD_-RIB5, P_GPD_-RIB7* | WT | “ |
| *A330* | *P_RIB7_-ADE12, P_GPD_-RIB1, P_GPD_-RIB2, P_GPD_-RIB3, P_GPD_-RIB5, P_GPD_-RIB7* | WT | “ |
| *A339* | *ade12Δ, P_GPD_-RIB1, P_GPD_-RIB2, P_GPD_-RIB3, P_GPD_-RIB5, P_GPD_-RIB7* | ade^-^ | “ |

Additional File - Table 1. *A. gossypii* strains used in this study.
