# Supplementary material for: Metabolic engineering of riboflavin production in Ashbya gossypii through pathway optimization
Source: Microb Cell Fact. 2015 Oct 14;14:163. doi: 10.1186/s12934-015-0354-x (PMC4605130; doi:10.1186/s12934-015-0354-x)
Supplement: Supplementary file 4 — 10.1186/s12934-015-0354-x List of primers used in this study. [file 12934_2015_354_MOESM4_ESM.docx]

Additional File - Table 2. List of primers used in this study.

| *Primer* | *Sequence* | *Purpose* |
| --- | --- | --- |
| P_GPD_-RIB1-ins5 | 5’-ATTTACCTTAGGCAAAAAGTAACAAAAGGCTTTTCCGTAGGTGCTTTGTCATTCAACAATCCAC GTCGGAATTGGCGACTATATAGTGTAGGGCCCATAAAGCAGTAGTCGGTGTTGACAGGGTAATATAGATCTGGTCG-3’ | *RIB1* overexpression |
| P_GPD_-RIB1-ins3 | 5’-TAGGTGTTCCTTGCTGTCGATCGAGTTGTGGTATAGATGGAGGAAGACATCGGTGCCCTGTA CCGTCGGTATGCGCGCGCGTGCGACACAGGTCACTTCTGGCACTGTGTATTCAGTCATTGTGCGGTGTGTATGTGTGG-3’ | *RIB1* overexpression |
| P_GPD_-RIB2-ins5 | 5’-ACTACGCCCCTACCATATTCGATCTTGTGGTATTGACGATATTCCTCTGTTTGGTTTTACTGG  CACTATTCCGTTTGACGGTATAGCGCTATTCGTTCATAGTGACACATGCGGCACACAGGGTAATATAGATCTGGTCG-3’ | *RIB2* overexpression |
| P_GPD_-RIB2-ins3 | 5’-CGAAGCGACCTGTCTTTTGCGACTCTCATCCTGCGATGTGTTTTCCATAATCCTAACCAGCCT  GGGTTTCAGATGACGTTGCGTCTCCTTAAAAAGAAGGCCAGGAACGCCTTTGAGCATTGTGCGGTGTGTATGTGTGG-3’ | *RIB2* overexpression |
| P_GPD_-RIB3-ins5 | 5’-GGTTGGGCTACCAAGGATAGTTGATGACTTCCATCACCTATAAAAGCGGCTTGAGTGCTTTT  GCAATGATTCTGTTCACATGATGGACAAGAAATACGTACAAAAATTTCAACGTTTACAGGGTAATATAGATCTGGTCG-3’ | *RIB3* overexpression |
| P_GPD_-RIB3-ins3 | 5’-CGCTGCTGCACATATTAGATCGGCCTCGTTTTCTCTCGAGATGTGGTCCATGACGATGATCAT  CTTATTTTGCTTGAACTGCTCTATAGCGGTACCGATATCAGTGCATGGGCTTGTCATTGTGCGGTGTGTATGTGTGG-3’ | *RIB3* overexpression |
| P_GPD_-RIB5-ins5 | 5’-AAAGTATAATCAGATAGTTAGTCGTATCTTCTAGTTTTATTAGTCAGCTACATGGCGAACCGCC  ATTTCCTTATGCATGTCTTACGAGTTTAAAAAGCTCGCGGTAGCAGAAAAGAAACAGGGTAATATAGATCTGGTCG-3’ | *RIB5* overexpression |
| P_GPD_-RIB5-ins3 | 5’-CGCCAGTATCGGAGCCGCATCCTTGATAAGGACTGACACACCGTTGCCGCCTGCCTCGCTG  GCATCGTTCTCCAAGTACTCAGCAACAGTGCCAATGTGTTCCACTATACCGGTAAACATTGTGCGGTGTGTATGTGTGG-3’ | *RIB5* overexpression |
| P_GPD_-RIB7-ins5 | 5’-TTCAAGCCATCTAAGGTACAGAAGCCCCAATTGAAGCGAACTGCATCGTCCCGGGCGGATG  AGAACGAGTTCTCGATATTATAGAGGCCCCCGTTTCGAGTGATTGGCGTCAAAAACACAGGGTAATATAGATCTGGTCG-3’ | *RIB7* overexpression |
| P_GPD_-RIB7-ins3 | 5’-ATCTAGGGACTGCGCATACGTCAGCGTGACAAACGGCAGGCGTGCGGATGAGTCCGGTGG  TGTCGGTAAGTACGGTGCTAGTATATCAGCCAGATCTTGAGAAAGTGGTATTAGCGCCATTGTGCGGTGTGTATGTGTGG-3’ | *RIB7* overexpression |
| ADE12-del5 | 5’-AGGCGTACTTTTAATGCTTTCGTTTCGCTTGGCAACGTAGTTGTATTACGTTCAGTATACAGG  CAGGCTAGCGTATATCCAGTCTCGGTCAGCACATATGCGGATCCCCGGGTTAATTAA-3’ | *ADE12* deletion |
| ADE12-del3 | 5’-GTACAAATAACACTTGTCTCGACTATTTTCAATCAATCTGCCTATTAGGTCTTTTATTTACCGC  TCACTACAAGTAGAGTTCCCAAGAGTCGGTCAACTAGAATTCGAGCTCGTTTAAAC-3’ | *ADE12* deletion |
| P_RIB7_-ADE12-ins5 | 5’-AATTAACCATTTGTGATAACATACAGATATATATATATATGTATTAATTTGAGGTCGCCTAGCT  ACTGGAAGGCGTACTTTTAATGCTTTCGTTTCGCTTGGCAACGTAGTTGTATTACAGGGTAATATAGATCTGGTCG-3’ | *ADE12* underexpression |
| P_RIB7_-ADE12-ins3 | 5’-GTGTCCAGCATTATTGCCCCCGGCGGACCGCGCTACAATGTCATACTTGCTTACCAGCAAAT  CCACGAGCTTGCCCTTACCTTCGTCACCCCACTGTGACCCTAGAACGACGTTGACCATCGAGTTCTCGATATTATAGAGGCCCC-3’ | *ADE12* underexpression |
| RIB1-RTfw | 5’-GCTCGCGGAGTCTGTTCCGG-3’ | *RIB1* qPCR |
| RIB1-RTrv | 5'-CACGCCCTGTGTGGGCTGAG-3' | *RIB1* qPCR |
| RIB2-RTfw | 5’-CGATGCCGATGAGGGCTCGC-3’ | *RIB2* qPCR |
| RIB2-RTrv | 5’-CCCGGTGGGATGGGCTGGAA-3’ | *RIB2* qPCR |
| RIB3-RTfw | 5’-AGCGCACATGACTGCCGAGC-3’ | *RIB3* qPCR |
| RIB3-RTrv | 5’-TGGCGGCAACTGCAGCCTTC -3’ | *RIB3* qPCR |
| RIB4-RTfw | 5’-GGCGAAGTTGATCAAACCTAC-3’ | *RIB4* qPCR |
| RIB4-RTrv | 5’-CCAAACTTTACAGCCATCTCC-3’ | *RIB4* qPCR |
| RIB5-RTfw | 5’-ATACTGGCGGATTGCCACAT-3’ | *RIB5* qPCR |
| RIB5-RTrv | 5’-ACTGTGCAACCTGCTTCTCT-3’ | *RIB5* qPCR |
| RIB7-RTfw | 5’-CCACCGGACTCATCCGCACG-3’ | *RIB7* qPCR |
| RIB7-RTrv | 5’-GCCGGCCGCACAAAACACTG-3’ | *RIB7* qPCR |
| ACT1-RTfw | 5’-TTTCGCCGGTGACGACGCTC-3’ | *ACT1* qPCR |
| ACT1-RTrv | 5’-CGTGTGGCAACGAGAAACCAGC-3’ | *ACT1* qPCR |
| GPD-RTfw | 5’-GATCAAGGTCTTCCAGGAGAG-3’ | *GPD* qPCR |
| GPD-RTrv | 5’-CTTGTTCAACTTGACGGTCAA-3’ | *GPD* qPCR |
